# Preparation of Tissues for Examination with the Microscope

**Published:** 1887-07

**Authors:** S. Flexner

**Affiliations:** Louisville


### The Preparation of Tissues for Examination with the Microscope with Special Reference to the Microscopical Examination of the Teeth.

BY S. FLEXNER, PH. G., OF LOUISVILLE.

Read before the Kentucky State Dental Society, June, 1887.

All objects require some preparation before they can be profitably examined with the microscope. The treatment necessary to secure satisfactory results varies within very wide limits, depending on the structure with which we are dealing and the result which it is desired to attain. In every case the method of preparation is based on a knowledge of the structure of the object intended for examination and the change which it undergoes when brought into contact with reagents whose properties are understood. In the application of reagents the greatest care and precision should always be observed lest we irreparably impair what otherwise may have been a valuable specimen. The intelligent and scientific preparation of tissues is therefore of the first importance, and I will endeavor to present in as short space as practicable those methods which are generally recognized as producing the best results, and which are endorsed by the most eminent microscopists. Objects intended for viewing with the microscope are mounted in one of two ways, as transparent objects or as opaque objects. The first is the method commonly applied to histological and pathological specimens, as it affords a view of the entire section, while the latter exhibits only the structure of the surface. To this end the first requisite is to secure a portion of the tissue so thin that light is readily transmitted through it and its structure discernible. This is accomplished either by teasing or section-cutting. By the first method the elements of the tissue separated under glycerine by needles are mounted in the same fluid and subjected to examination, while by the second thin even sections are cut by means of a razor, the tissue having been previously prepared to fit it for this operation. Teasing is applicable to fresh material, while section cutting is not, unless indeed one is possessed of a freezing microtome.

As it is not always convenient to prepare and examine specimens as they are received, the preservation of them for future examination and study is an important matter. As this is involved in and elucidated under the subject of hardening specimens, it will be dismissed here. Of the processes for preparing tissues for examination section-cutting is to be recommended. When it is desired not so much to preserve specimens as to make rapid diagnoses, teasing will answer a good purpose, but the patience requisite to secure satisfactory results, and the disarrangement of the parts of the structure which inevitably ensues are considerable limitation to its employment. Therefore the cutting of the sections is preferable, particularly when it is desirable to preserve the specimens.

Ordinarily, indeed in every case where the freezing microtome is not employed, the tissue (if it is soft) has first to be hardened. This is accomplished by means of agents which act by coagulating the albumen and gelatine, present in the tissues, and by the abstraction of water. The substances most commonly employed are alcohol, Muller's fluid, chromic, acid and glycerine.

The desideratum in the hardening of tissues is to obtain the desired result with as little shrinkage as possible. The reason for this is evident, and should ever be in mind when attending to this part of the process. Hardening should be done slowly and with precision. No one method can be applied to all tissues, but each agent has its special applications. I will enumerate them in the order in which they are generally employed.

1st. Alcohol hardens by coagulating albumen and gelatine and abstracting water. Tissue to be hardened in it, should be cut in pieces a cubic inch in size and passed through successive solutions at intervals of three days having different strengths, as follows: 45ʒ 60ʒ 80ʒ 95ʒ. Almost all kinds of tissue may be hardened in alcohol, the exceptions being embryonic tissue, myxomatta, and callow tumors.

2d. Muller's fluid hardens by coagulating albumen and gelatine, but abstracting but little water. Causing little or no shrinkage, it is of great applicability, and its use is attended with little risk to the tissues. It acts slowly and should be renewed often. The pieces immersed in it should be of the size of those hardened in alcohol. If at the end of four or six weeks the specimen is not sufficiently hardened, it should be transferred through the different strengths of alcohol enumerated before, to 95 alcohol for completion. The time for immersion in each strength is to be much shortened. Muller's fluid is prepared as follows: Bichromate potash, two parts; sulphate sodium, one part; water, one hundred parts; mix and dissolve.

3d. Chromic acid is used in solutions containing $\frac{1}{2}$ percent. It acts like Muller's fluid, but more energetically This solution must be renewed every few days, but it will do in days what Muller's fluid takes weeks to accomplish.

4th. Glycerine acts wholly by attracting water, and should be diluted at first, the pieces immersed being small. Thin sections of the hardened tissue are made by holding the tissue in the hand and cutting with a razor (free hand sections) and imbedding in paraffine or wax, and cutting with a microtome (mechanical sections). The staining of the sections follows the cutting. Probably no greater progress has been made in any department of biological science than in the staining of tissue for the purpose of differentiation. Staining depends on the affinity of different elements of one structure for special stains or different amounts of one stain. A given tissue may be made to take one stain in such a way as to show the different parts by the degree to which each is stained; or it may be stained with reference to bringing out one part with one color and other parts with other colors—double and treble staining. A great variety of stains are in use, of which I will mention a few: Ammonia carmine, alum carmine, borax carmine, picro carmine, indigo carmine, hæmatoxylon, and the aniline stains in general. 1st. The ammonia carmine, which has given the best results in my hands, is Beale's. It is made as follows: Carmine, 10 grs.; strong water of ammonia, $\frac{1}{2}$ drachm; glycerine and water, of each 2 ounces; alcohol $\frac{1}{2}$ ounce. Dissolve the carmine in the ammonia by the aid of heat and leave the solution exposed for one hour for the excess of ammonia to evaporate, then add the other ingredients and filter.

2d. Alum carmine, Grenacher's formula, is as follows: To 100 parts of 5 percent solution of pure alum add 1 part of carmine; boil for twenty minutes; allow to cool and filter.

3d. Borax carmine. Take of carmine, 30 grains; borax, 2 drachms; distilled water, 4 ounces; mix and decant; do not filter. Stain for a few minutes and wash in muriatic acid 1 part, alcohol 20 parts, till the section assumes a bright rose tint.

4th. Picro carmine, Ranvier's formula, is satisfactory. It is as follows: Rub 1 gramme carmine with 10 c. c. of distilled water, and add 3 c. c. of aq. ammonia. Add to this 200 c. c. of a cold saturated solution of picric acid. Evaporate slowly in a water bath to one-third at a low temperature. This is a double stain.

5th. Indigo carmine. This stain is used in conjunction with borax carmin for double staining.

6th. Logwood is very useful in many cases. It brings out nuclei quite as well as carmine, and makes a valuable contrast stain. With picro carmine and eosin it double stains effectively.

7th. Aniline stains. These are various and very beautiful. They do not differentiate as well as some mentioned, but are useful in special cases. It remains to mention nitrate silver, chloride gold, and osmic acid. These are only used in special cases, and need not be dwelt on here.

To stain successfully, practice is required. Little excepting general rules can be obtained from books. A few failures may be the means of making the whole procedure intelligible if the causes are carefully sought out.

The mounting of the section is next to be accomplished. Some workers regard this step as indifferent, at least in point of importance. Perhaps this is one reason that so many cabinets on examination are found to contain a great number, if not a predominating number, of poor and useless specimens. For my part I take great pride in this operation. The mounting fluids are reducible into resinous and aqueous media. In this latter division I include glycerine. The type of the former is Canada Balsam. This is variously prepared for use. It may be employed as it is or concentrated—that is, with its oil driven off and dissolved in Benzole, xylol, alcohol, chloroform, etc. For usual operations I prefer the Benzole Balsam. It has the merit of drying quite rapidly and of being easily soluble in benzine. This I consider important, as the cleaning of the slide can be done with this cheap agent. Glycerine is an excellent mounting medium, and is preferred by such an eminent microscopist as Lionel Beale. Mounts in glycerine have to be cemented, as the glycerine does not dry, and is apt to absorb moisture. Further, this is essential to prevent the cover glass from being displaced. The use of round covers makes this easy if one is possessed of a turn-table. Gold size I find admirable for this purpose. It dries by oxidation, becoming insoluble, and is therefore not acted on by the glycerine, even after a long time. Mixtures of gum, water, and glycerine have been recommended as self-drying fluids. I have had so little experience with them that I am not prepared to say with what success they may be used. Before mounting in balsam clarification of the section is necessary. This is done usually with oil of cloves. If Benzole Balsam is used the clarifying may be done with benzole. Glycerine mounts become clear through the actions of the medium itself.

### THE MICROSCOPICAL EXAMINATION OF TEETH.

Teeth resembling bone in many particulars require much the same treatment before they are ready for microscopical examination. To examine them in situ it is best to take the lower jaw of some small animal, as a rat or mole, and decalcify it with chromic and hydrochloric acids. After the lime salts are washed out it is to be cut into sections and the sections stained in picro carmine and logwood and mounted in Canada balsam. To prepare sections of non-decalcified teeth sawing and grinding is the accepted method. Much time may be saved in this operation if we provide ourselves with a couple of emery wheels, one moderately coarse and one very fine, and use a lathe. For vertical sections the tooth is sawed into halves and the flat edges smoothed on the fine wheel. They are then cemented to a glass slide with Canada balsam and the grinding done first with the coarse, and then with the fine wheel, until the desired thinness is obtained. Transverse sections are sawed and finished in the same general way as vertical, only a greater number can be made from one tooth. By warming the balsam the sections are removed and are then ready for permanent mounting. Dentine—the components are a firm matrix, dentinal canals, and dentinal fibres. To see the fibres magnifying powers of 500 to 1,000 diameters are required. The dentinal sheaths the lining of the tubules can best be seen in cross sections, when they appear as delicate yellowish rings, in the interior of which the transverse section of the dentinal fibre is perceptible in the form of a minute dark spot.

The dentinal tubules are best shown in sections dried in air. They then appear in the form of strongly defined very dark tubules or lines. Concentrated nitric acid brings out the dentinal canals. The fibres easily stain with carmine.

Enamel.—If young teeth are examined at the stage when they are capable of being cut with the knife the enamel prism may be clearly observed. Transverse sections exhibit a delicate net work with six sided areas. In the adult the enamel prisms are demonstrable after treatment first with dilute hydrochloric acid, and afterwards with sulphuric acid.

The Cuticula.—The structure of this is demonstrated after its removal from young teeth with hydrochloric acid just as they are perforating the gum. It is stained in nitrate of silver.

Cement.—In its chemical and microscopical character it is closely allied to bone. It calls for the same treatment.

The Pulp.—In many respects it recalls in structure the mucous tissue of old umbilical cord. Consisting of finely fibrous connective tissues liberally supplied with cells, it is treated similarly to soft structures from other parts. Its cavernous appearance is given it by the capillary vessels it contains. I hope with the assistance of Dr. Hooper, to exhibit to you to night specimens which will illustrate the different processes mentioned in this paper. In this connection I desire to express my thanks to Dr. Hooper for his valuable and cordial assistance.

---

Dr. Davenport, state analyst of Massachusetts, has examined twenty advertised cures for the opium-habit, and found that all but one contained opium. This one was called " double chloride of gold," but contained no trace of gold.

